# Heat shock protein 27 regulates myogenic and self-renewal potential of bovine satellite cells under heat stress

**DOI:** 10.1093/jas/skad303

**Published:** 2023-09-09

**Authors:** Won Seob Kim, Jayasimha R Daddam, Boon Hong Keng, Jaehwan Kim, Jongkyoo Kim

**Affiliations:** Department of Animal Science, Michigan State University, East Lansing, MI 48824, USA; Department of Animal Science, Michigan State University, East Lansing, MI 48824, USA; Department of Food Science and Human Nutrition, Michigan State University, East Lansing, MI 48824, USA; Department of Animal Science, Michigan State University, East Lansing, MI 48824, USA; Department of Animal Science, Michigan State University, East Lansing, MI 48824, USA; Department of Food Science and Human Nutrition, Michigan State University, East Lansing, MI 48824, USA; Animal Science and Food Science and Human Nutrition, Michigan State University, East Lansing, MI 48824, USA

**Keywords:** bovine satellite cells, heat shock proteins, heat stress, myogenic regulatory factors

## Abstract

While satellite cells play a key role in the hypertrophy, repair, and regeneration of skeletal muscles, their response to heat exposure remains poorly understood, particularly in beef cattle. This study aimed to investigate the changes in the transcriptome, proteome, and proliferation capability of bovine satellite cells in response to different levels of heat stress (**HS**) and exposure times. Satellite cells were isolated from 3-mo-old Holstein bulls (body weight: 77.10 ± 2.02 kg) and subjected to incubation under various temperature conditions: 1) control (38 °C; **CON**), 2) moderate (39.5 °C; **MHS**), and extreme (41 °C; **EHS**) for different durations ranging from 0 to 48 h. Following 3 h of exposure to extreme heat (EHS), satellite cells exhibited significantly increased gene expression and protein abundance of heat shock proteins (**HSPs**; HSP70, HSP90, HSP20) and paired box gene 7 (Pax7; *P* < 0.05). HSP27 expression peaked at 3 h of EHS and remained elevated until 24 h of exposure (*P* < 0.05). In contrast, the expression of myogenic factor 5 (**Myf5**) and paired box gene 3 (Pax3) was decreased by EHS compared to the control at 3 h of exposure (*P* < 0.05). Notably, the introduction of HSP27 small interference RNA (siRNA) transfection restored Myf5 expression to control levels, suggesting an association between HSP27 and Myf5 in regulating the self-renewal properties of satellite cells upon heat exposure. Immunoprecipitation experiments further confirmed the direct binding of HSP27 to Myf5, supporting its role as a molecular chaperone for Myf5. Protein–protein docking algorithms predicted a high probability of HSP27–Myf5 interaction as well. These findings indicate that extreme heat exposure intrinsically promotes the accumulation of HSPs and modulates the early myogenic regulatory factors in satellite cells. Moreover, HSP27 acts as a molecular chaperone by binding to Myf5, thereby regulating the division or differentiation of satellite cells in response to HS. The results of this study provide a better understanding of muscle physiology in heat-stressed cells, while unraveling the intricate molecular mechanisms that underlie the HS response in satellite cells.

## Teaser

This study explores how satellite cells in beef cattle respond to heat stress (**HS**), revealing significant changes in gene expression and protein levels, including increased heat shock proteins and decreased myogenic factor 5 (**Myf5**) and paired box gene 3 (Pax3) expression. It is concluded that this study reveals that heat shock protein 27 (HSP27) acts as a molecular chaperone, which interacts with Myf5, clarifying on how satellite cells respond to HS and better understanding the physiology of muscles in heat-stressed cells.

## Introduction

Livestock production in the modern era has encountered substantial economic losses due to the impact of global climate change. With climate change, the frequency of sudden temperature extremes is expected to rise, leading to an increased risk of fatalities among animals unaccustomed to heat stress (**HS**) and resulting in significant financial setbacks for the beef cattle industry (St-Pierre et al., 2003; Wall et al., 2010). Among meat-producing animals, beef cattle are particularly susceptible to ambient and radiant heat extremes as they are primarily raised outdoors. Furthermore, many preferred cattle breeds in the United States possess black coats, which further amplifies heat absorption.

Animals within the thermal neutral temperature range are capable of maintaining core temperatures with minimal metabolic regulation, remaining in a state of comfort and requiring no additional energy expenditure to maintain body temperature (American Dairy Science Association, 2020). Temperatures exceeding this range enter the HS zone, demanding additional efforts from animals such as increased energy expenditure and behavioral modifications to sustain body homeostasis. Hyperthermia disrupts the delicate balance between metabolic heat production and dissipation, leading to alterations in animal metabolism and cellular functions. Rapid changes in body temperature trigger the release of catecholamines from endocrine organs (Aggarwal and Upadhyay, 2013), subsequently impairing the cellular immune response (Bagath et al., 2019).

One of the crucial cellular responses during thermal stress involves the activation of transcription and accumulation of heat shock proteins (**HSPs**) (De Maio, 1999). These HSPs facilitate cellular adaptation to environmental changes and play a significant role in adapting to environmental stress and maintaining a balance, especially in relation to protein function (Kim et al., 2020a). Additionally, HSPs are associated with the development of thermotolerance and the rapid restoration of heat-induced denatured proteins (Maloyan et al., 1999). Especially among these HSPs, HSP27 plays an important role in skeletal muscle development. Previous studies have demonstrated that HSP27 is expressed during critical stages of muscle differentiation (Dubińska-Magiera et al., 2014; Kim et al., 2018a). Additionally, it has been revealed that HSP27 functions as a target gene for transcription factor myocyte enhancer factor 2 (**MEF2**), which controls the advanced phases of myogenesis (Huang et al., 2007). Huang et al. (2007) assume that MEF2 factor working in coordination with myoblast determination protein 1 (**MyoD**) and other myogenic regulatory factors (**MRFs**), could potentially contribute to upregulation of HSP27 in differentiated skeletal muscles.

Satellite cells, located adjacent to skeletal muscle fibers, serve as stem cells responsible for muscle tissue growth, repair, and regeneration. During postnatal stages, satellite cells undergo proliferation and fuse with preexisting muscle fibers, contributing to the increase in number of myonuclei and muscle hypertrophy (Pallafacchina et al., 2013). Despite limited knowledge about the effects of HS on hypertrophy and satellite cell activity in skeletal muscles, previous research has yielded inconsistent findings. Some studies report detrimental effects of heat exposure during muscle growth, while others indicate potential benefits. Previous studies have yielded different results due to various factors, such as the type of livestock, experimental design, growth period, and cell culture conditions. Especially, studies investigating the response to HS at the cellular level and animal models can also yield different results. For instance, Ma et al. (2018) observed decreased breast muscle yield in broiler chickens exposed to 32 °C for 14 d, which downregulated insulin-like growth factor 1 (IGF-1), IGF-1 receptor, mechanistic target of rapamycin (**mTOR**), and myogenin gene expressions.

Oxidative damage to muscles due to HS has also been reported in swine (Montilla et al., 2014 [35 °C for 1 or 3 d]; Ganesan et al., 2017 [37 °C for 12 h]; Pardo et al., 2021 [30 °C for 30 d]) and poultry (Mujahid et al., 2007 [34 °C for 18 h]; Pamok et al., 2009 [38 °C for 21 d]). Conversely, other studies suggest that HS can increase skeletal muscle hypertrophy through satellite cell proliferation and protein synthesis (Goto et al., 2003 [41 °C for 96 h]; Uehara et al., 2004 [41 °C for 1 h]; Kojima et al., 2007 [41 °C for 1 h]). In rodent and turkey studies, HS was found to enhance skeletal muscle size via the mTOR signaling pathway (Oishi et al., 2009 [42 °C for 1 h]; Takeuchi et al., 2014 [42 °C for 20 min]; Xu et al., 2022 [43 °C for 72 h]). In swine, HS (40.5 °C for 48 h) altered microRNA related to HS and increased protein turnover (Kamanga-Sollo et al., 2011). Additionally, increased proliferation and differentiation of satellite cells have been observed in humans and turkey (Yamaguchi et al., 2010 [39 or 41 °C for 72 h]; Clark et al., 2017 [43 °C for 72 h]; Xu et al., 2021b [43 °C for 72 h]). Despite the abundance of research on other meat-producing species, only a few studies have investigated the effects of heat exposure on tissue growth in beef cattle. Reith et al. (2022) conducted a study to investigate the transcriptomic responses of beef steers exposed to zilpaterol hydrochloride and HS (temperature–humidity index 73 to 85 for 21 d). Their findings revealed that HS influenced pathways associated with oxidative stress, and the use of beta-adrenergic agonists exacerbated this negative effect. Another consequence of HS is its impact on protein synthesis and catabolism in skeletal muscle (Baumgard and Rhoads, 2013).

As previously mentioned, satellite cells play a crucial role in determining the rate and extent of postnatal muscle growth by providing the necessary nuclei for muscle fiber development. Understanding the effects of HS on cell proliferation and MRFs is vital for comprehending its impact on muscle physiology and postnatal development. Therefore, in this study, we hypothesize that heat exposure regulates satellite cell activity by modulating the expression of HSPs and myogenic regulatory signals. Our objectives include examining the effects of heat exposure on bovine satellite cells (**BSCs**) and predicting its potential consequences on postnatal skeletal muscle development.

## Materials and Methods

### BSC isolation

Cell isolation protocol for this project (PROTO202000294) was reviewed and approved by the Michigan State University Institutional Animal Care and Use Committee (IACUC). BSCs were isolated from 3-mo-old Holstein bull calves (*n* = 3, body weight: 77.10 ± 2.02 kg). The calves were harvested at the Michigan State University Meat Laboratory (East Lansing, MI, USA) under USDA inspection. The isolation and cultivation protocol for BSCs followed the methods described in previous studies (Johnson et al., 1998; Kim et al., 2018b). Longissimus muscle tissue was collected using a sterile knife and immediately transported to the Michigan State University Muscle Biology Laboratory (East Lansing, MI, USA). Under aseptic conditions, the muscle was processed to remove blood vessels, connective tissue, and adipose tissue. Subsequently, the muscle was passed through a sterile meat grinder. The ground muscle was incubated with 0.1% of Pronase (Calbiochem, LaJolla, CA, USA) and Earl’s Balanced Salt ­Solution (EBSS; Sigma-Aldrich, St. Louis, MO, USA) in a shaking water bath for 1 h at 37 °C. After incubation, the mixture was centrifuged at room temperature at 1,500 × *g* for 4 min. The supernatant was discarded, and the resulting pellet was resuspended in phosphate-buffered saline (**PBS**; Sigma-Aldrich). The resuspended cells were centrifuged at room temperature at 500 × *g* for 10 min, and the supernatant was removed. This process was repeated, resulting in a pellet of mononucleated cells.

### BSC culture and HS conditions

Cells were plated and incubated in Dulbecco’s Modified Eagle’s medium (**DMEM**; Gibco, Waltham, MA, USA) supplemented with 10% fetal bovine serum (**FBS**; Thermo Fisher Scientific) and 1× Antibiotic–Antimycotic (Gibco) at 38 °C under a humidified atmosphere of 95% O_2_ and 5% CO_2_. Serial passages were performed to purify the cells based on their confluency. Cells at the exponential phase with passage numbers 2 to 4 were used for all experiments.

**Figure 1.**
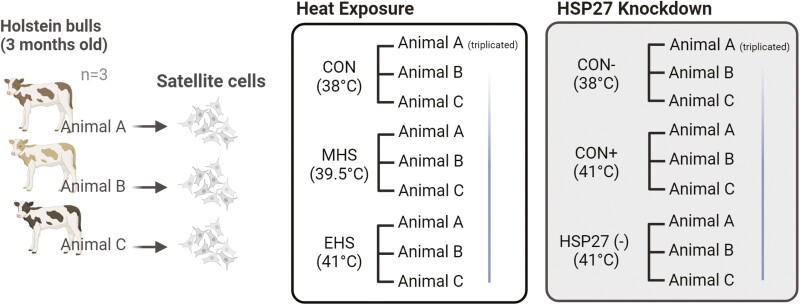
Experimental design schematic. In this study, each subject animal serves as a unique and independent unit (Animal A, B, and C) of replication. Sequential measurements are undertaken for each subject to foster robust data acquisition, thereby augmenting the statistical validity and reliability of the research findings. Created with BioRender.com.

The BSCs were seeded on to either 96-well or 6-well plates at a density of 1 × 10^4^ or 1 × 10^5^ cells/mL respectively, in DMEM supplemented with 10% FBS and 1× Antibiotic–Antimycotic (Gibco) at 38 °C under a humidified atmosphere of 95% O_2_ and 5% CO_2_. In addition, for proteomic analysis, the BSCs were seeded on a T75 flask at a density of 1 × 10^5^ cells/mL under the same conditions. The cells were incubated at 38 °C for 24 h to allow for cell attachment. After 24 h, cells were divided into three temperature groups: 38 °C (control; **CON**), 39.5 °C (moderate heat stress; **MHS**), and 41 °C (extreme heat stress; **EHS**). The treatments were further divided into seven subgroups (0, 1, 3, 6, 12, 24, and 48 h) based on the incubation time, with three replications (Figure 1). For proteomic analysis, only the 3-h treatment was conducted, with three replications. The experimental cells were placed in another incubator set to the required experimental temperature, while the control cells were maintained at 38 °C. The cell culture media was changed every 24 h throughout the experiment in each group.

### Small interference RNA treatment

The specific small interference RNAs (**siRNAs**) targeting HSP27 (Dharmacon, Lafayette, CO, USA) were used for gene knockdown in BSCs. Both HSP27-specific siRNAs were mixed in equal proportions to create a siRNA cocktail (Table 1). The transfection of siRNAs into BSCs was performed using Lipofectamine 2000 transfecting reagent (50 nM, Thermo Fisher Scientific, Waltham, MA, USA). The transfection efficiency was optimized by testing different concentrations ranging from 50 to 100 nM. A negative control siRNA (50 nM, Dharmacon) was included as a control group.

**Table 1.** Sequences of siRNA selected to target the bovine HSP27 gene (NM_001025569.1)

Name^1^	Sequence	Length
HSP27 1 Sense (5ʹ–3ʹ)	UGA AAC ACC GCC UGC UAA AUU	21
HSP27 1 Antisense (5ʹ–3ʹ)	UUU AGC AGG CGG UGU UUC AUU	
HSP27 2 Sense (5ʹ–3ʹ)	AGG ACG GCG UGG UGG AGA UUU	21
HSP27 2 Antisense (5ʹ–3ʹ)	AUC UCC ACC ACG CCG UCC UUU	

*^1^HSP27*, heat shock protein 27; siRNA, small interference RNA.

Following transfection, the cells were incubated for 24 h, after which the growth media (DMEM with 10% FBS) was changed. After another 24 h, the cells were divided into three temperature groups: CON (−) (38 °C), CON (+) (41 °C), and HSP27 (−) (41 °C) for a duration of 3 h (Figure 1).

### Proliferation assay

Cell proliferation was assessed using the Cell Counting Kit-8 (Abcam, Cambridge, United Kingdom) with five replicates per run. BSCs were seeded at a density of 1 × 10^4^ cells/mL in 96-well plates and incubated under temperature treatment conditions for various time intervals including 0, 1, 3, 6, 12, 24, and 48 h. Following the heat treatments, 10 µL of the Cell Counting Kit-8 solution was added to each well, and the cells were further incubated at their respective treatment temperatures for 3 h. Subsequently, the absorbance was measured at 460 nm using a SpectraMax M5 microplate reader (Molecular Devices, Silicon Valley, CA, USA). Cell proliferation was calculated using the following formula: *cell proliferation = optical density (OD) (sample) − OD (blank).*

### mRNA extraction and real-time quantitative polymerase chain reaction

RNA extraction was performed via TRIzol reagent (Invitrogen, Carlsbad, CA, USA) modified from previous method of Kim et al. (2018b). The concentration and purity of mRNA were determined with a spectrophotometer at absorbances of 260 nm and 280 nm using a NanoDrop One/One^C^ Microvolume UV-Vis Spectrophotometer (Thermo Fisher Scientific). An acceptable range for the 260/280 ratio was 1.78 to 1.91. To remove genomic DNA and synthesize complementary DNA (**cDNA**), the QuantiTect reverse transcription kit (Qiagen, Germantown, MD, USA) was utilized following the manufacturer’s instructions. Real-time quantitative polymerase chain reaction (**PCR**) was conducted using the QuantStudio 6 Pro system (Applied Biosystems) to determine the gene expression levels relative to *ribosomal protein subunit 9 (****RPS9***) and *hydroxymethylbilane synthase (****HMBS***) mRNA in total RNA. *RPS9* and *HMBS* are commonly used as housekeeping genes due to their consistent expression across bovine tissues (Bionaz and Loor, 2007; Janovick-Guretzky et al., 2007; Pérez et al., 2008; Kim et al., 2018b). The Ct values of these housekeeping genes remained unchanged across the heat treatment group in the current study. Therefore, *RPS9* and *HMBS* were employed as endogenous controls to normalize the expression of the target genes. The relative quantification of the target cDNA was assessed using TaqMan Fast Advanced Master Mix (Applied Biosystems) and TaqMan Gene Expression Assays (Thermo Fisher Scientific) (Table 2). Triplicate assays were performed following the manufacturer’s recommended thermal cycling parameters, consisting of 45 cycles of 15 s at 95 °C and 1 min at 60 °C. All real-time PCR reactions and Ct value analyses were conducted using the QuantStudio 6 Pro System from Applied Biosystems.

**Table 2.** TaqMan probes and primers used for RT-qPCR assays

Gene^1^	TaqMan probe assay	Target gene	Manufacturer
*RPS9*	Bt03272016_m1		Thermo Fisher
*HMBS*	Bt03234763_m1		
*HSP20*	Bt03213719_m1	Crystallin alpha B (CRYAB)	
*HSP27*	Bt03220563_m1	Heat shock protein family B (small) member 1	
*HSP70*	Bt03292670_g1	Heat shock protein family A member 1A	
*HSP90*	Bt03244099_g1	Heat shock protein 90 alpha family class B member 1	
*MyoD*	Bt03244740_m1		
*Myf5*	Bt03223134_m1		
*MyoG*	Bt03258928_m1		
*Pax3*	Bt04303789_m1		
*IGF1*	Bt03252282_m1		

Gene	Primer and probe sequence (5ʹ–3ʹ)		Manufacturer
*Pax7*			Thermo Fisher
Forward	GCCCTCAGTGAGTTCGATTAG		
Reverse	GATGCTGTGCTTGGCTTTC		
Probe	6FAM-TTCGTCCTCCTCCTCCTTCTTCCC-MGBNFQ		

^1^ *RPS9*, ribosomal protein subunit 9; *HMBS*, hydroxymethylbilane synthase; *HSP*, heat shock protein; *MyoD*, myoblast determination protein 1; *Myf5*, myogenic factor 5; *MyoG*, myogenin; *Pax3*, paired box gene 3; *Pax7*, paired box gene 7; *IGF1*, insulin-like growth factor.

### Immunofluorescence microscopy

Cells were seeded on 4-well Lab-Tek chamber slides (Thermo Fisher Scientific) and incubated at 38 °C for 24 h to promote cell adhesion. Subsequently, the slides were fixed with 4% paraformaldehyde (Thermo Fisher Scientific) for 15 min at room temperature. After three washes with PBS, the cells were permeabilized with 0.1% Triton X-100 (Thermo Fisher Scientific) in PBS for 15 min. To minimize nonspecific binding, a blocking solution containing 2% bovine serum albumin (Thermo Fisher Scientific) in PBS was applied and incubated for 1 h at 4 °C.

The cells were then incubated overnight at 4 °C with the following primary antibodies: anti-paired box gene 7 (**Pax7**; mouse monoclonal, diluted 1:500, PAX7, Developmental Studies Hybridoma Bank [**DSHB**], Iowa City, IA, USA), HSP27 (rabbit polyclonal, diluted 1:500; bs-0730R, Bioss Antibodies, Inc.), and anti-HSP70 (rabbit polyclonal, diluted 1:500; bs-5365R-A680, Bioss Antibodies, Inc.). After three washes with PBS, the cells were incubated with the secondary antibody Alexa Fluor 488 anti-mouse IgG (1:1,000; ab2536161, Thermo Fisher Scientific) for 30 min at room temperature. F-actin was labeled with Texas Red-X ­Phalloidin (1:1,000; T7471, Thermo Fisher Scientific). Subsequently, the slides were washed three times with PBS and counterstained with 4ʹ6-diamidino-2-phenylindole (**DAPI**, 1:1,000; 62248, Thermo Fisher Scientific) in PBS for 5 min at room temperature. Finally, coverslips were mounted onto glass slides using Fluoromount-G Mounting Medium (Thermo Fisher Scientific) and sealed with nail polish. The slides were imaged using fluorescent microscopy (EVOS M5000, Thermo Fisher Scientific) at a magnification of 20×. To determine the populations of Pax7-positive cells, five random images were captured from each slide, and the quantities of DAPI- and Pax7-stained nuclei were averaged (Supplementary Figure 1).

### Western blotting

At the 3-h time point of HS treatment, lysates were prepared from satellite cells using mammalian protein extraction reagent (M-PER) lysate buffer (Thermo Fisher Scientific) supplemented with protein inhibitor (Thermo Fisher Scientific). The total protein content in the lysates was determined using the bicinchoninic acid (**BCA**) assay (Thermo Fisher Scientific) with a NanoDrop One/One^C^ Microvolume UV-Vis Spectrophotometer (Thermo Fisher Scientific), measuring the absorbance at 562 nm. For protein analysis, 20 µg of protein samples were denatured at 70 °C for 10 min followed by 85 °C for 2 min and loaded onto a Bolt 4% to 12% gradient Bis-Tris Plus gel (Thermo Fisher Scientific). Electrophoresis was performed at 200 V for 22 min, and the proteins were subsequently transferred onto a nitrocellulose membrane using the iBlot 2 Dry Blotting System (Thermo Fisher Scientific). To block nonspecific binding, the membranes were incubated with iBind Flex Solution (iBind Flex Buffer, Thermo Fisher Scientific) for 10 min at room temperature. Primary antibodies including anti-HSP70 (mouse monoclonal, dilution 1:1,000; Ab53496, Abcam Inc., Cambridge, MA, USA), anti-HSP27 (mouse monoclonal, dilution 1:1,000; CPTC-HSPB1-1, DSHB), anti-phospho-HSP27 (rabbit polyclonal, dilution 1:500, ab5581, Abcam Inc.), anti-Pax7 (mouse monoclonal, dilution 1:1,000; PAX7, DSHB), anti-myogenic factor 5 (**Myf5**; rabbit polyclonal, dilution 1:1,000; ab125301, Abcam Inc.), anti-MyoD (rabbit monoclonal, dilution 1:1,000; D8G3, Cell Signaling, Danvers, MA, USA), and glyceraldehyde 3-phosphate dehydrogenase (**GAPDH**) (mouse monoclonal, dilution 1:1,000; DSHB-hGAPDH-2G7, DSHB) were incubated with the membranes. Secondary antibodies, including Goat anti-mouse IgG H&L (horseradish peroxidase [**HRP**]) (mouse polyclonal, dilution 1:1,000; ab205719, Abcam Inc.) and Goat anti-rabbit IgG H&L (HRP) (rabbit polyclonal, dilution 1:2,000; ab205718, Abcam Inc.), were applied using the iBind Flex Western System (Thermo Fisher Scientific) at room temperature for 4 h. The membranes were then incubated with the SuperSignal West Pico Chemiluminescent Substrate (Thermo Fisher Scientific) and the protein bands were visualized using a FluorChem imager (Alpha Innotech, San Leandro, CA, USA).

### Immunoprecipitation

At the 3-h time point of HS treatment, cultured BSCs were harvested using 150 µL of NP-40 lysis buffer (Thermo Fisher Scientific) with protein inhibitor (Thermo Fisher Scientific). The protein content of the samples was quantified using the BCA protein assay kit (Thermo Fisher Scientific). For immunoprecipitation (**IP**), anti-HSP27 (5 µg, DSHB), normal rabbit serum (Invitrogen), and Protein A-Sepharose 4B (Invitrogen) were added to the cell lysate, followed by an overnight incubation at 4 °C. The bead–antibody–antigen complex was then centrifuged at 4 °C for 1 min, and the supernatant was discarded. The complex was washed three times for 1 min each with NP-40 buffer at 4 °C. After washing, 50 µL of sample buffer was added, and the mixture was boiled at 95 °C for 10 min. The samples were subsequently analyzed by western blotting. For western blotting, anti-Myf5 (Abcam) and Goat anti-rabbit IgG H&L (HRP) were used at a dilution of 1:1,000.

### Sample preparation for proteomics analysis

Protein samples (50 µg) (*n* = 3/each group) were precipitated with trichloroacetic acid (**TCA**) and subjected to tandem mass spectrometry (MS/MS) quantification at the Michigan State University Proteomics Core. The TCA-precipitated pellets were resuspended in 270 µL of 100 mM ammonium bicarbonate supplemented with 4% sodium deoxycholate (**SDC**). To reduce and alkylate the samples, tris(2-carboxyethyl)phosphine (TCEP) and chloroacetamide were added at concentrations of 10 mM and 40 mM, respectively. The mixture was incubated for 5 min at 45 °C with shaking at 2,000 rpm using an Eppendorf ThermoMixer. Trypsin was added at a ratio of 1:100 (wt/wt) in 50 mM ammonium bicarbonate, and the digestion was performed overnight at 37 °C with shaking at 1,500 rpm in the ThermoMixer. The final volume of each digest was approximately 300 µL. Following digestion, SDC was removed through phase extraction. The samples were acidified with 1% trifluoroacetic acid (**TFA**) and subjected to C18 solid-phase cleanup using StageTips to eliminate salts.

### Liquid chromatography with tandem mass spectrometry and data analysis

Samples were resuspended in 20 µL of 2% acetonitrile (**ACN**)/0.1% TFA. An injection of 2 µL was automatically made using a Thermo EASYnLC 1200 (Thermo Fisher Scientific) onto a Thermo Acclaim PepMap RSLC 0.075-mm × 500-mm C18 column and washed for ~5 min with buffer A. Bound peptides were then eluted over 65 min with a gradient of 8%B to 38%B in 54 min, ramping to 90%B at 55 min and held at 90%B for the duration of the run (Buffer A = 99.9% Water/0.1% Formic Acid; Buffer B = 80% ACN/0.1% Formic Acid/19.9% Water) at a constant flow rate of 300 nL/min. The column temperature was maintained at a constant temperature of 50 °C using an integrated column oven (PRSO-V2, Sonation GmbH, Biberach, Germany). Elute peptides were sprayed into a ThermoScientific Q-Exactive HF-X mass spectrometer (Thermo Fisher Scientific) using a FlexSpray spray ion source. Survey scans were taken in Orbitrap (60,000 resolution, determined at m/z 200) (Shalit et al., 2015). The top 15 ions in each survey scan were then subjected to automatic higher energy collision-induced dissociation (HCD) with fragment spectra acquired at 15,000 resolutions.

The raw data obtained was processed, and protein measurements were conducted using intensity-based label-free proteomics, following a modified method based on Shalit et al. (2015). The resulting MS/MS spectra were converted into peak lists using MaxQuant v1.6.3.4 (www.maxquant.org) (Cox and Mann, 2008) and subsequently searched against a database of all available Bovine protein sequences from Uniprot (www.uniprot.org), supplemented with common laboratory contaminants. The Andromeda search algorithm, integrated into the MaxQuant environment and based on the modified method by Cox et al. (2011), was employed for the search. The Mascot output was further analyzed using Scaffold, v5.1.2 (www.proteomesoftware.com), to validate protein identifications probabilistically. Protein assignments that passed the Scaffold 1% FDR (false discovery rate) confidence filter were considered valid. The specific peptides of interest, namely HSP20, HSP27, HSP70, HSP90, Myf5, and Pax7, were focused to determine any changes in order to corroborate the findings from the western blot.

### Interaction prediction modeling of HSP27 and Myf5

The sequences of HSP27 and Myf5 from *Bos taurus* was collected using the UniProt database (www.uniprot.com) were submitted to Basic Local Alignment Search Tool (BLAST) program (http://www.ncbi.nlm.nih.gov) to search for a template to be used to develop structures against the Protein Data bank database (https://www.rcsb.org). In the absence of these structures for the selected sequences, prediction modeling methods were used to build a three-dimensional model using the Modeller program. The interactions between HSP27 and Myf5 from *Bos taurus* molecules were predicted using H-DOCK (http://hdock.phys.hust.edu.cn). To investigate the HSP27–Myf5 mechanism, the predicted models were submitted to the H-DOCK server, and binding sites were selected based on the analysis from the Castp server. The interaction between HSP27 and Myf5 was determined, and the amino acids involved in the interaction were visualized using Discovery Studio and Molegro Visualizer software. Furthermore, the LigPlot+ software was utilized to generate visual representations of the amino acids involved in the interactions.

### Statistical analysis

Data analysis was performed using GraphPad Prism Version 9.4.1 (Graph Pad Software, San Diego, CA, USA). The significance of HS (CON, MHS, and EHS), time (0, 1, 3, 6, 12, 24, and 48 h), and their interaction for cell proliferation and gene expression were determined using two-way analysis of variance (**ANOVA**) followed by the Tukey’s honest significant difference (**HSD**) test. Proteomics data, after logarithmic transformation, were also analyzed by two-way ANOVA and *t*-test to determine the effects of HS treatment: CON vs. MHS, CON vs. EHS. Differentially expressed proteins for each effect were determined at *P* < 0.05. Gene expression and protein levels at the specified time point (3 h) were analyzed by one-way ANOVA, followed by Tukey’s HSD post hoc comparisons. Data are shown as standard errors of means. An α level of 0.05 was used to determine significance, with tendencies discussed at *P*-values between 0.05 and 0.10.

## Results

### Satellite cell population

The satellite cell population in primary culture was identified by Pax7 immunofluorescence, a method commonly used to identify satellite cells (Feng et al., 2018; Jung et al., 2022). On this basis, 96.6% of cell population appeared to be Pax7-positive (Supplementary Figure 1).

### Heat shock proteins

**Figure 2.**
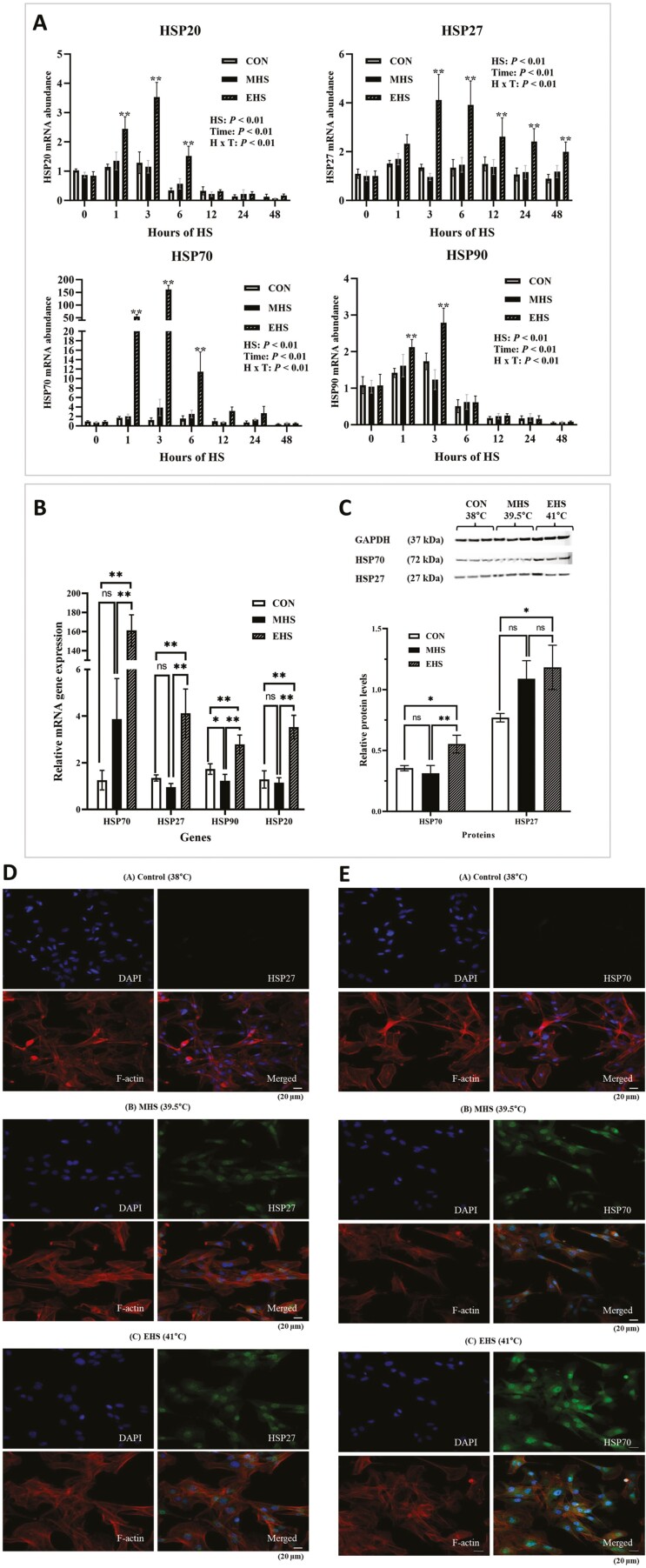
Heat shock proteins (HSPs) expression was elevated during heat exposure. (A) *HSP20*, *27*, *70*, and *90* mRNA levels in CON, MHS, and EHS for 48 h. All HSPs peaked (*P* < 0.05) at 3 h after heat exposure in EHS. (B) *HSP20*, *27*, *70*, and *90* mRNA levels in CON, MHS, and EHS for 3 h. (C) Western blots (top) and quantification of protein levels (bottom) of *HSP27* and *HSP70* in CON, MHS, and EHS for 3 h. (D, E) Immunohistochemistry of *HSP27* and *HSP70* expression in BSCs for 3-h heat exposure. Nuclei were stained with 4ʹ6-diamidino-2-phenylindole (DAPI, blue). *HSP27* and *HSP70* were stained with a rabbit polyclonal anti-*HSP27*, *HSP70* (green). F-actin was visualized with Texas Red-X Phalloidin (red). Scale bars, 20 µm. All data are presented as mean ± standards errors (*n* = 9). The *P*-values are determined by two-way ANOVA (A) or one-way ANOVA (B, C) (*P < 0.05, **P < 0.01). ANOVA, analysis of variance; BSCs, bovine satellite cells; CON, control; EHS, extreme heat stress; MHS, moderate heat stress.

An interaction between the time of heat exposure and the temperatures was noted (*P* < 0.01) for mRNA expression of *HSP20*, *27*, *70*, and *90* (Figure 2A). The main effect of heat exposure altered the expression of all HSP genes. Cells exposed to EHS induced expressions of all HSP isoforms, with peaks at 3 h after heat exposure (Figure 2B). Expression of *HSP20*, *70*, and *90* gradually decreased after 6 h of heat exposure and returned to the baseline. However, *HSP27* expression in cells was maintained up to 48 h after EHS exposure. As all measured HSPs peaked 3 h after heat exposure, we chose the 3-h time point of heat exposure as an optimal time window to determine further gene expressions and protein levels. Cells exposed to EHS for 3 h significantly increased (*P* < 0.01) expression of *HSP70* and *27* compared to CON and MHS cells. The protein abundance of HSP70 was also the greatest (*P* < 0.01) in EHS cells (Figure 2C–E). HSP27 protein level was increased (*P* < 0.05) in EHS-exposed satellite cells compared to CON. However, no difference was observed with MHS. The gene expression of *HSP90* was increased (*P* < 0.01) in EHS but decreased in MHS (*P* < 0.05) compared to CON. *HSP20* was the greatest (*P* < 0.01) in cells exposed to EHS. *HSP70* was most responsive to heat exposure and showed a 128.2-fold increase compared to CON.

### HS and cell proliferation

**Figure 3.**
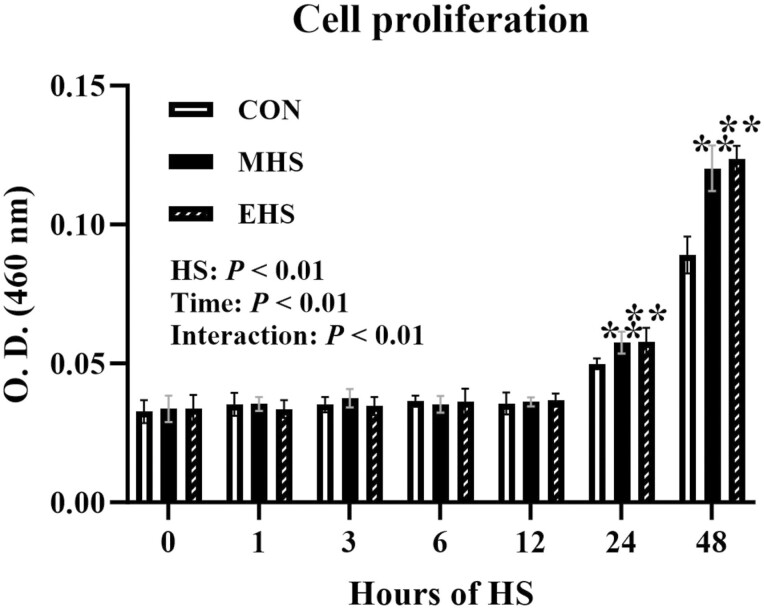
Effect of heat stress on cell proliferation in primary bovine satellite cells. Following the heat treatment for 48 h, the cells were further incubated at their respective treatment temperatures for 3 h. The data are presented as mean ± standard errors (*n* = 9). The *P*-values are determined by two-way ANOVA (**P < 0.01). ANOVA, analysis of variance.

A temperature and time interaction (*P* < 0.01) was found for the satellite cell proliferation rate. In response to both MHS and EHS of exposure, the cell proliferation rate was increased (*P* < 0.01) after 24 h and 48 h compared to CON (Figure 3).

### Myogenic regulatory factors

**Figure 4.**
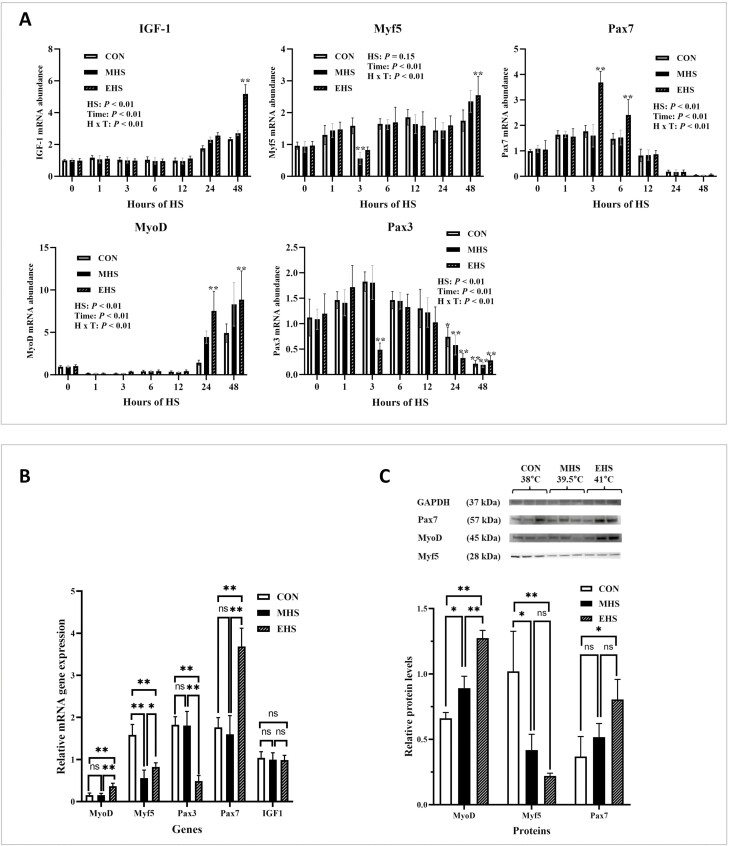
Myogenic regulatory factors were altered during heat exposure. (A) *MyoD*, *Myf5*, *IGF1*, *Pax3*, and *Pax7* mRNA levels in CON, MHS, and EHS for 48 h. (B) *MyoD*, *Myf5*, *IGF1*, *Pax3*, and *Pax7* mRNA levels in CON, MHS, and EHS for 3 h. (C) Western blots (top) and quantification of protein levels (bottom) of *MyoD*, *Myf5*, and *Pax7* in CON, MHS, and EHS for 3 h. All data are presented as mean ± standard errors (*n* = 9). The *P*-values are determined by two-way ANOVA (A) or one-way ANOVA (B, C) (*P < 0.05, **P < 0.01). ANOVA, analysis of variance; CON, control; EHS, extreme heat stress; MHS, moderate heat stress.

To evaluate the impact of HS on factors associated with myogenic regulation, we investigated the expression of two paired-box transcription factors, paired box gene 3 (**Pax3**) and Pax7. Our results revealed significant interactions between exposure time and temperatures for all examined genes (*P* < 0.01). Specifically, the expression of *Pax3* was downregulated (*P* < 0.01) after 3 h of exposure to EHS, while no significant differences were observed at other sampling times. In contrast, cells exposed to EHS for 3 and 6 h exhibited increased expression of *Pax7* (Figure 4A and B). Furthermore, the protein abundance of Pax7 was also elevated (*P* < 0.05) following EHS exposure (Figure 4C). However, after 12 h of exposure, the *Pax7* mRNA expression returned to levels similar to CON and MHS conditions. Notably, the expression of both *Pax3* and *Pax7* decreased over time (*P* < 0.01). We also examined other early MRFs, namely Myf5 and MyoD. MHS and EHS treatments led to decreased mRNA expression of *Myf5* after 3 h of exposure (Figure 4B). Additionally, the protein abundance of Myf5 was reduced by both MHS and EHS treatments (Figure 4C). When cells were exposed to MHS and EHS for more than 24 h, the expression of *MyoD* was increased (*P* < 0.01) (Figure 4A). Specifically, the gene expression of *MyoD* was elevated in cells exposed to EHS (*P* < 0.01) for 3 h (Figure 4B), while both MHS (*P* < 0.05) and EHS (*P* < 0.01) conditions resulted in increased protein abundance of MyoD compared to CON (Figure 4C). Among the different treatments, cells exposed to EHS for 3 h exhibited the highest protein abundance of MyoD (*P* < 0.01) (Figure 4C).

### HSP27 knockdown

**Figure 5.**
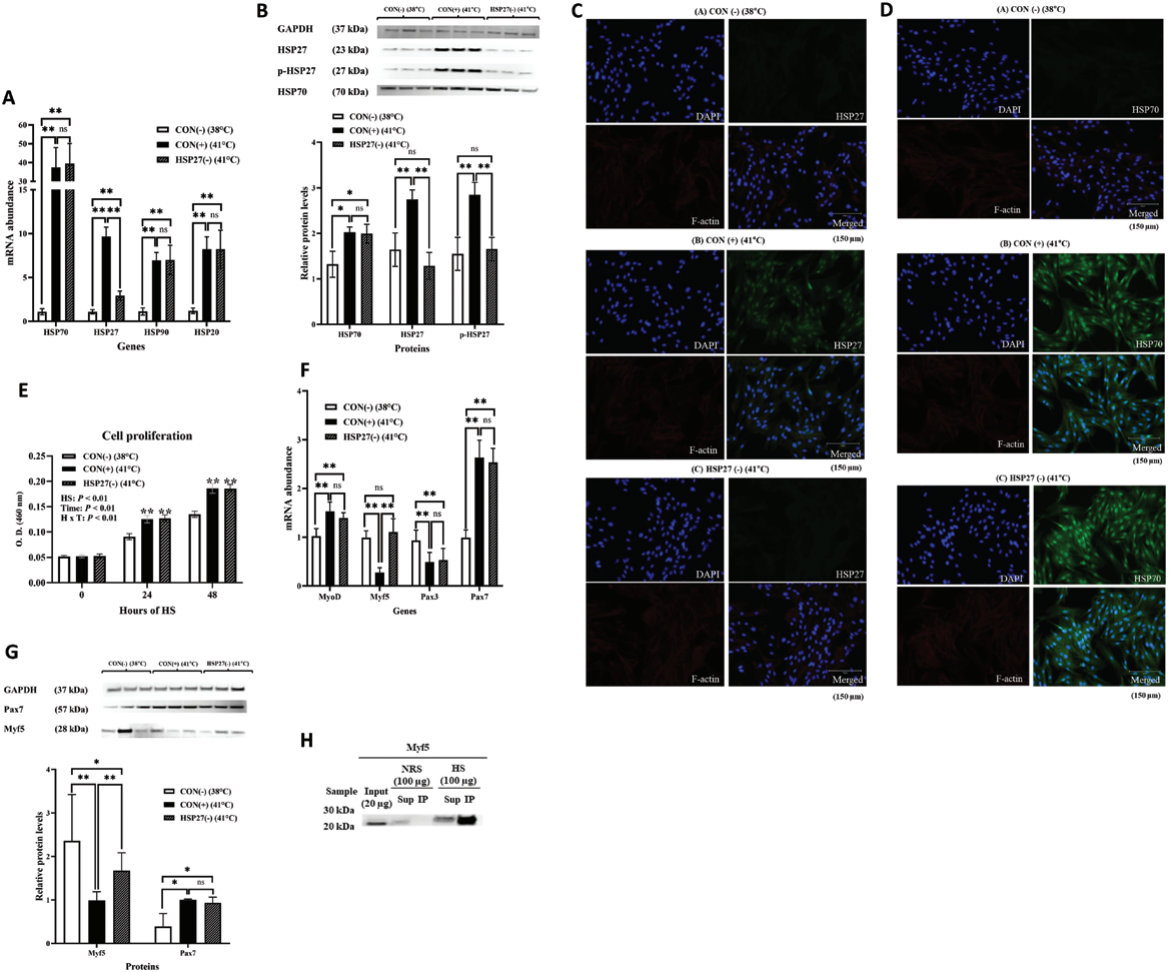
Heat shock protein (HSP) 27 knockdown on HSPs and myogenic regulatory factors expression and cell proliferation during heat exposure. (A) *HSP20*, *27*, *70*, and *90* mRNA levels in CON (−), CON (+), and HSP27 (−) for 3 h. (B) Western blots (top) and quantification of protein levels (bottom) of *HSP27* and *HSP70* in CON (−), CON (+), and HSP27 (−) for 3 h. (C, D) Immunohistochemistry of *HSP27* and *HSP70* expression in *HSP27* knockdown BSCs for 3-h heat exposure. Nuclei were stained with 4ʹ6-diamidino-2-phenylindole (DAPI, blue). *HSP27* and *HSP70* were stained with a rabbit polyclonal anti-*HSP27*, *HSP70* (green). F-actin was visualized with Texas Red-X Phalloidin (red). Scale bars, 150 µm. € Cell proliferation in CON (−), CON (+), and HSP27 (−) for 48 h. Following the heat treatment for 48 h, the cells were further incubated at their respective treatment temperatures for 3 h. (F) *MyoD*, *Myf5*, *Pax3*, and *Pax7* mRNA levels in CON (−), CON (+), and HSP27 (−) for 3 h. (G) Western blots (top) and quantification of protein levels (bottom) of *Myf5* and *Pax7* in CON (−), CON (+), and HSP27 (−) for 3 h. (H) Immunoprecipitation (IP) with anti-*HSP27* antibodies followed by western blotting with anti-*Myf5* antibodies. Input is a positive control, NRS (normal rabbit serum) is a negative control, IP is the target. All data are presented as mean ± standard errors (*n* = 9). The *P*-values are determined by one-way (A, B, F, G) or two-way (E) ANOVA (*P < 0.05, **P < 0.01). ANOVA, analysis of variance; CON, control.

In order to investigate the effects of depleting HSP27, satellite cells were transfected either with a negative control siRNA or HSP27 siRNA in either sense or antisense orientation. Following transfection, the cells were subjected to EHS for 3 h. Transfection of BSCs with *HSP27* siRNA significantly reduced (*P* < 0.01) the expression of *HSP27* by approximately 70%. However, the expression of other HSP isoforms (*HSP70*, *HSP90*, and *HSP20*) remained unchanged (Figure 5A) between CON (+) (41 °C) and HSP27 (−) (41 °C). Furthermore, the protein abundance of HSP27 and phosphor HSP27 were decreased by approximately 53.3% and 41.9% (*P* < 0.01), while HSP70 levels were not affected by siRNA treatment (Figure 5B). The knockdown of HSP27 was also confirmed through immunohistochemical analysis (Figure 5C and D). A temperature and time interaction (*P* < 0.01) was found for the transfected cell proliferation rate. In response to both CON (+) (41 °C) and HSP27 (−) (41 °C) of exposure, the cell proliferation rate was increased (*P* < 0.01) after 24 h and 48 h compared to CON (−) (38 °C). However, the depletion of HSP27 did not alter the cell proliferation during heat exposure (Figure 5E). The depletion of HSP27 did not alter the mRNA expression of *MyoD*, *Pax3*, and *Pax7* (Figure 5F). However, the expression of *Myf5* returned to control levels, which was consistent with the observations at the protein level (Figure 5G). To investigate the protein–protein interaction between HSP27 and Myf5, an IP assay and western blot were performed. The IP test revealed that HSP27 binds to Myf5 under HS conditions (Figure 5H).

### Proteomic analysis

Using an untargeted approach, within the proteome of the BSCs exposed to either MHS or EHS, 2,065 raw reads were matched and identified with 1,523 unique proteins. Altogether, only 80 peptides in CON vs. MHS treatment and 41 peptides in CON vs. EHS treatment were differentially expressed (*P* < 0.05) (Supplementary Table 1). HSP70 (fold change [**FC**] = 2.5) and HSP90 (FC = 1.3) were both significantly upregulated (*P* < 0.05) in EHS conditions, reflecting the results seen previously. However, this change was not seen in cells exposed to MHS (*P* > 0.05). Other HSPs such as HSP20 and HSP27 were not significant as well in both MHS and EHS conditions (*P* > 0.05). Furthermore, MRFs and paired-box transcription factors, such as Pax3 and Pax7, were not observed/detected from the proteomic analysis from the cells in both MHS and EHS conditions.

### Modeling of interactions between HSP27 and Myf5

**Figure 6.**
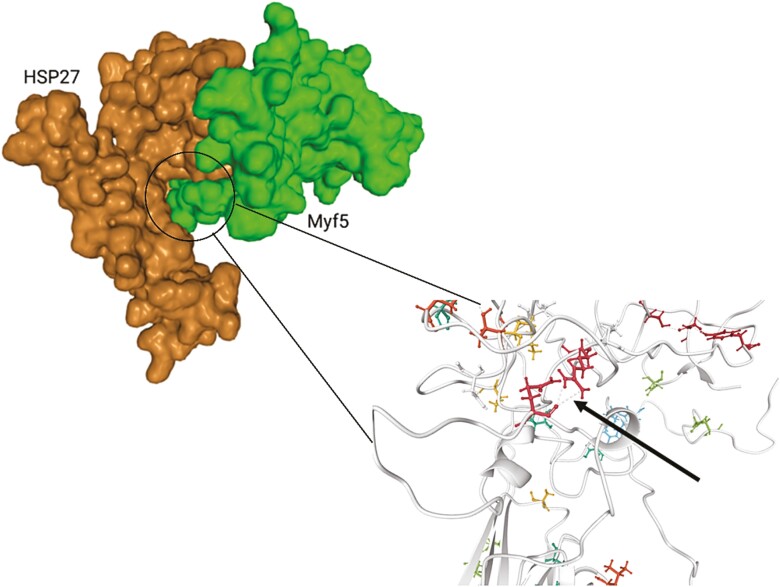
In silico model prediction of possible protein–protein interactions of *HSP27* and *Myf5* using docking algorithms (H-DOCK). 3d protein-protein interaction model was created from HDOCK (http://hdock.phys.hust.edu.cn/) and BioRender.com.

The molecular docking of HSP27 and Myf5 elucidated a substantial likelihood of interaction between these entities. Primarily, upon the binding of HSP27 to Myf5, a discernible interaction between these protein counterparts was observed (Figure 6). Furthermore, it was observed that the residues within HSP27 established a robust hydrogen bond with Myf5, thus signifying the potential for a strong interaction. The specific bonding interactions were emphasized and visualized using the LigPlot+ software (Figure 6).

## Discussion

Heat exposure cues substantial protein cascades and alters transcriptional signaling pathways in various cells as part of cytoprotection (Collier et al., 2008). HSPs accumulate within cells immediately after exposure to stressors such as heat, hypoxia, metabolic stress, high-strengthening exercise, and infections (Lee et al., 2007). The HSPs function as molecular chaperones that help maintain homeostasis and prevent damage to cells (Mosser et al., 1997; Kregel, 2002; Kim et al., 2020b). Particularly in skeletal muscle, HSPs are reported to be involved in the synthesis of new proteins, stabilizing native protein structure, reducing damage, and repairing damaged proteins (Koh, 2002; Liu et al., 2006). However, the regulatory role of HSPs on satellite cells under HS is still unclear. Given the importance of satellite cells to postnatal myofiber hypertrophy and regeneration, it is imperative to understand the cellular mechanisms that take place under the influence of HS regulated by HSPs.

We confirmed that satellite cells exposed to EHS immediately increased mRNA gene expression and protein abundance of *HSP70*, *90*, *27*, and *20*. The expression of *HSP70* was the most responsive to temperature changes in BSCs, which showed 128-fold changes than CON. Our data are consistent with those of other studies in human and rodent models (Lepore et al., 2001; Liu et al., 2006). At 3 h, *HSP70* gene expression peaked during EHS exposure and returned to the CON level after 12 h. This suggests that the *HSP70*’s function in the cells is augmented after 3 h of expression and possibly diminishes over time. Protective functions of *HSP70* in skeletal muscle exposed to stressors have been discussed previously and include protection against muscle damage, promotion of muscle regeneration and recovery, and maintenance of skeletal muscle mass and integrity (Senf et al., 2013). In response to stress, HSP70 has been argued to play an important role in regulating cell proliferation against short heat exposure (Gao et al., 2015). However, the regulatory mechanism of *HSP70* in satellite cell activation is still poorly understood.

We also confirmed that EHS increased the gene expression and protein level of *HSP90*. Notably, HSP90 appears to play a crucial role in myogenic differentiation and acts as a mediator for satellite cell fusion. Wagatsuma et al. (2011) reported that *HSP90* inhibition downregulated the expression of the MRFs *MyoD*, myogenin (***MyoG***), and sarcomeric myosin heavy chain C2C12 cells.

In addition, our investigations revealed a distinct pattern for *HSP27* gene expression. Following exposure to EHS, the gene expression of *HSP27* reached its peak at 3 h and remained elevated for 48 h. Conversely, other HSPs returned to normal levels after 6 h. HSP27 is widely distributed in ­various tissues and is recognized as a versatile chaperone (Collier and Benesch, 2020). Unlike some other HSPs, small HSPs do not require adenosine triphosphate for activation (Dubińska-Magiera et al., 2014). Its ability to interact with various proteins suggests involvement in multiple cellular processes.

In the process of myogenic differentiation of satellite cells, HSP27 assumes a regulatory role in determining the fate of satellite cells, whether they generate daughter cells capable of self-renewal or myogenic progenitors. This regulation occurs through its interaction with other MRFs, primarily mediated by p38 Mitogen-activated protein kinase (MAPK) signaling (Segalés et al., 2016; Ding et al., 2018). Our present findings provide evidence suggesting that HSP27 may possess a critical function in governing the dynamics of satellite cells and the process of myogenic differentiation under conditions of HS.

When cells were exposed to EHS for a duration of 3 h, we observed an increase in *Pax7* gene expression, while *Pax3* and *Myf5* exhibited a decrease. Satellite cell activity is controlled by multiple transcription factors, among which the *Pax3* and *Pax7* play significant roles. These transcription factors, along with MyoD, Myf5, and myogenin, collectively known as MRFs, contribute to the regulation of satellite cell activity (Relaix et al., 2006). Both Pax3 and Pax7 are paired-box transcription factors presumed to be essential in myogenesis, whether during the developmental phase or in adult skeletal muscle. Pax7, as well as its paralogue Pax3, are present in quiescent satellite cells as well as in actively proliferating cells. Although these transcription factors exhibit overlapping roles in regulating early myogenesis, Pax3 is selectively active in embryonic muscle precursors, while Pax7 governs both fetal and early postnatal muscle development (Wang and Rudnicki, 2012).

Satellite cells constitute a heterogeneous population, consisting of satellite stem cells characterized by Pax7 expression and lack of Myf5 (Pax7+/Myf5−) and satellite myogenic cells expressing both Pax7 and Myf5 (Pax7+/Myf5+). Pax7 plays a crucial role in satellite cell proliferation and in promoting myogenic precursors that express Myf5 (Wang and Rudnicki, 2012). Our data demonstrated a notable alteration in the satellite cell population exposed to heat, resulting in a shift from Pax7+/Myf5+ to Pax7+/Myf5−. This change suggests that under HS conditions, satellite cells exhibit a preference for maintaining a proliferative or quiescent state rather than undergoing myogenic differentiation. Consequently, this may account for the observed increased proliferation rate in cells exposed to MHS and EHS. Similar enhancements in proliferation have been previously reported in turkey satellite cells (Clark et al., 2016; Xu et al., 2021a) and swine satellite cells (Kamanga-Sollo et al., 2011). Although some studies claimed that the HS condition could lead to the arrest of the cell cycle to avoid damage to cellular molecules, thus inhibiting cell proliferation (Kim et al., 2020a; Zhou et al., 2020), this may vary depending on the cell source and conditions. In the case of animal studies, HS can mediate immune response and metabolism. Given that in vitro setting does not involve other factors such as immune responses or hormonal regulations, any increase in cell proliferation could be solely attributed to the effects of heat exposure.

The precise mechanisms by which small HSPs contribute to postnatal skeletal muscle function and satellite cell activity in the presence of HS remain incompletely understood, and there exist contradictory findings in this regard. Initial investigations conducted on Sprague–Dawley rats indicated that the buildup of HSP25 and HSP72 is likely to impede muscle hypertrophy by diminishing muscle protein content and suppressing Type I muscle fibers (Frier and Locke, 2007). In contrast, Huang et al. (2021) reported that *HSP27* is a target gene for the transcription of *MEF2* regulating advanced stages of myogenesis. The previous embryonic fibroblast study found a positive correlation between *HSP27* expression and muscle growth. The expression of *HSP27* decreased, and muscle development and protein synthesis decreased in bovine embryonic fibroblast cells (Kim et al., 2018b). It was also found that HSP27 expression was increased after myogenic differentiation in the bovine skeletal muscle (Zhang et al., 2010). According to previous studies and our findings, HSP27 may impact satellite cell activity and alter the signaling cascades that are directly impacted by heat exposure.

Differentially expressed proteins from the proteomic data were shown in Supplementary Table 1 but none of the changes of the key transcription factors in this study such as Myf5, Pax3, and Pax7 were observed/detected within the proteomic data set. This may be due to transcription factors being relatively low in expression when compared to other proteins and that a proper methodology design should be considered prior to proteomic analysis. Additionally, this being a discovery-based study of different HS conditions on BSCs, the approach was not carefully specified nor considered for the targeting on the differences of MRFs. The difficulty of the detection and subsequent changes in transcription factors were expressed by Simicevic and Deplancke (2017). The only caveat was the increased in HSP70 for cells exposed to EHS conditions, which reflected both western blot and immunostaining results. Using our results, for example, only with a large FC change in mRNA expression of HSP70 indicated a small FC in proteomic analysis, thus confirming the mentioned difficulty. The selection of utilizing a proteomic analysis may not have been the optimum way to detect the abundant changes of the key transcription factors due to the possibility of posttranslational modifications as well. Rather, utilizing an alternative method such as a phosphor-proteomic approach should be considered. Comparative phosphor-proteomics and proteomics profiling done by Xiao et al. (2022) showed the differences of abundance for each method in differentiating murine myoblasts. Future plans would involve the use of such techniques to enrich and confirm the results obtained from the western blot. Additionally, utilizing high-throughput transcriptional techniques such as RNA-seq would provide useful verification of these transcription factors being present.

As noted earlier, mRNA gene expression and protein level of *Myf5* were decreased in cells exposed to heat. The *HSP27* siRNA knockdown resulted in the return of *Myf5* expression and protein abundance to the CON levels during heat exposure. Because *HSP27* siRNA transfection did not alter other markers including, *MyoD*, *Pax3*, or *Pax7*, HSP27 was thought to regulate Myf5 exclusively. The results of the IP assay suggest that HSP27 binds to Myf5 and controls the activities of satellite cells dependent on Pax7 under the influence of HS. The connection between HSP27 and Myf5 bears great importance, particularly in the context of satellite cell proliferation where Myf5 and Pax7 have an established relationship. Using protein–protein in silico docking studies, we also predicted the possible interaction between HSP27 and Myf5 using H-DOCK. The presence of a hydrogen bond between HSP27 and Myf5 provides evidence of interactions, where the ability of the binding site allocation and optimal reduction of steric hindrance (Luo et al., 2010) increases the probability of interaction between the two proteins, further supporting the results of the IP assay.

The current study demonstrated the intricate interplay between HSP27 and Myf5, a pivotal regulator in myogenic processes. Functioning as a chaperone protein, HSP27 assumes a pivotal role in regulating the transcriptional processes of Myf5. Additionally, the current study highlights that the knockdown of HSP27 leads to the restoration of Myf5 expression. These findings suggest a dual role for HSP27—acting not only as a chaperone to protect against HS but also as a significant regulator in muscle growth by influencing Myf5 transcription.

## Conclusion

The study suggests that heat exposure can enhance satellite cell proliferation, but the specific effects may vary depending on the cell source and conditions. The precise mechanisms by which HSPs contribute to skeletal muscle function and satellite cell activity during HS require further investigation.

*HSP27* was found to bind to *Myf5* and regulate satellite cell activities dependent on *Pax7* under HS conditions. Overall, these findings contribute to our understanding of the complex cellular responses to HS and highlight the potential role of HSPs, particularly *HSP70*, *HSP90*, and *HSP27*, in modulating satellite cell dynamics and myogenic differentiation during heat exposure.

It is important to note that the present study exclusively focused on heat exposure, while acknowledging that various factors, including the immune system and hormones, likely contribute to the modulation of cellular function and characteristics in animals. Further investigations conducted in vivo settings are necessary to validate the response of satellite cells to HS in animal models.

## Supplementary Material

skad303_suppl_Supplementary_Figure

skad303_suppl_Supplementary_Table

## Abbreviations

ACN: acetonitrile
ANOVA: analysis of variance
BCA: bicinchoninic acid
BSCs: bovine satellite cells
cDNA: complementary DNA
CON: control
DAPI: 4ʹ6-diamidino-2-phenylindole
DMEM: Dulbecco’s Modified Eagle’s medium
DSHB: Developmental Studies Hybridoma Bank
EHS: extreme heat stress
FBS: fetal bovine serum
FC: fold change
GAPDH: glyceraldehyde 3-phosphate dehydrogenase
HMBS: hydroxymethylbilane synthase
HRP: horseradish peroxidase
HS: heat stress
HSD: honest significant difference
HSP: heat shock protein
IP: immunoprecipitation
MEF2: myocyte enhancer factor 2
MHS: moderate heat stress
MRF: myogenic regulatory factor
mTOR: mechanistic target of rapamycin
Myf5: myogenic factor 5
MyoD: myoblast determination protein 1
MyoG: myogenin
Pax3: paired box gene 3
Pax7: paired box gene 7
PBS: phosphate-buffered saline
PCR: polymerase chain reaction
RPS9: ribosomal protein subunit 9
SDC: sodium deoxycholate
siRNA: small interference RNA
TCA: trichloroacetic acid
TFA: trifluoroacetic acid
