# Supplementary material for: Heat shock protein 27 regulates myogenic and self-renewal potential of bovine satellite cells under heat stress
Source: J Anim Sci. 2023 Sep 9;101:skad303. doi: 10.1093/jas/skad303 (PMC10629447; doi:10.1093/jas/skad303)
Supplement: skad303_suppl_Supplementary_Figure [file skad303_suppl_supplementary_figure.docx]

**
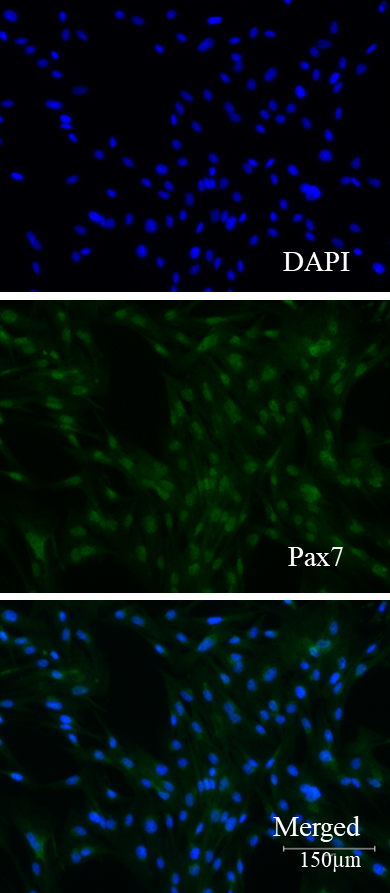
**

**Supplement Figure 1. Immunohistochemistry of Pax7 expression in primary bovine satellite cells.** Nuclei were stained with 4′6-diamidino-2-phenylindole (DAPI, blue). Pax7 was stained with a mouse monoclonal anti- Pax7 (green).
