# Supplementary material for: Heat shock protein 27 regulates myogenic and self-renewal potential of bovine satellite cells under heat stress
Source: J Anim Sci. 2023 Sep 9;101:skad303. doi: 10.1093/jas/skad303 (PMC10629447; doi:10.1093/jas/skad303)
Supplement: skad303_suppl_Supplementary_Table [file skad303_suppl_supplementary_table.docx]

**Supplement Table 1. Identified peptides using an untargeted approach within the proteome of the primary bovine satellite cells exposure heat stress. A** 80 peptides (42 up-regulated and 38 down-regulated peptides) were differentially expressed (*P* < 0.05) in mild heat stress (MHS)-exposed satellite cells compared to control (CON). **B** 41 peptides (22 up-regulated and 19 down-regulated peptides) were differentially expressed (*P* < 0.05) in extreme heat stress (EHS)-exposed satellite cells compared to control (CON). The up-regulated peptide expressed a positive fold change, and the down-regulated peptide expressed a negative fold change.

| **A** | |  | | | | |
| --- | --- | --- | --- | --- | --- | --- |
| **No** | **Protein** | | **Accession Number** | **Alternate ID** | **Log2**  **(Fold change)** | **T-TEST**  **(*P*-value)** |
| 1 | 26S proteasome regulatory subunit 10B OS=Bos taurus OX=9913 GN=PSMC6 PE=3 SV=2 | | F1MLV1 | PSMC6 | -0.86 | 0.00054 |
| 2 | Cluster of Calpain-2 catalytic subunit OS=Bos taurus OX=9913 GN=CAPN2 PE=2 SV=2 (Q27971) | | Q27971 | CAPN2 | 0.76 | 0.00096 |
| 3 | Pyrroline-5-carboxylate reductase 3 OS=Bos taurus OX=9913 GN=PYCR3 PE=2 SV=1 | | Q58D08 | PYCR3 | 1.04 | 0.0019 |
| 4 | Aconitate hydratase, mitochondrial OS=Bos taurus OX=9913 GN=ACO2 PE=1 SV=1 | | A0A3Q1M6K6 | ACO2 | -0.34 | 0.0021 |
| 5 | Cluster of 60S ribosomal protein L5 OS=Bos taurus OX=9913 GN=RPL5 PE=1 SV=1 (A0A3Q1MMW5) | | A0A3Q1MMW5 | RPL5 | 0.87 | 0.0029 |
| 6 | Heteroous nuclear ribonucleoprotein D like OS=Bos taurus OX=9913 GN=HNRNPDL PE=1 SV=1 | | A0A3Q1NLI5 | HNRNPDL | -0.43 | 0.0032 |
| 7 | CHMP4B protein OS=Bos taurus OX=9913 GN=CHMP4B PE=1 SV=2 | | Q08E32 | CHMP4B | 1.76 | 0.0036 |
| 8 | RNA-binding motif protein, X chromosome OS=Bos taurus OX=9913 GN=RBMX PE=1 SV=1 | | D3JUI8 | RBMX | -1.35 | 0.0041 |
| 9 | Cluster of Non-POU domain containing octamer binding OS=Bos taurus OX=9913 GN=NONO PE=2 SV=1 (Q2KJ42) | | Q2KJ42 | NONO | 1.40 | 0.0048 |
| 10 | Cluster of Ras-related protein Rab-18 OS=Bos taurus OX=9913 GN=RAB18 PE=4 SV=1 (A0A452DIB1) | | A0A452DIB1 | RAB18 | -0.72 | 0.0053 |
| 11 | Cluster of Glioblastoma amplified sequence OS=Bos taurus OX=9913 GN=NIPSNAP2 PE=2 SV=1 (Q3SWX4) | | Q3SWX4 | NIPSNAP2 | 0.87 | 0.0066 |
| 12 | Prosaposin OS=Bos taurus OX=9913 GN=PSAP PE=4 SV=1 | | A0A3Q1MI01 | PSAP | 0.88 | 0.0091 |
| 13 | Non-muscle caldesmon OS=Bos taurus OX=9913 GN=CALD1 PE=1 SV=2 | | F1MLW0 | CALD1 | 0.87 | 0.0096 |
| 14 | THO complex subunit 2 OS=Bos taurus OX=9913 GN=THOC2 PE=3 SV=3 | | F1N153 | THOC2 | 1.67 | 0.01 |
| 15 | 116 kDa U5 small nuclear ribonucleoprotein component OS=Bos taurus OX=9913 GN=EFTUD2 PE=3 SV=1 | | A0A3Q1MII4 | EFTUD2 | 3.66 | 0.011 |
| 16 | Eukaryotic translation initiation factor 5 OS=Bos taurus OX=9913 GN=EIF5 PE=3 SV=1 | | F1N0F7 | EIF5 | -0.96 | 0.011 |
| 17 | Cluster of Eukaryotic translation initiation factor 1 OS=Bos taurus OX=9913 GN=EIF1 PE=3 SV=1 (Q5E938) | | Q5E938 | EIF1 | -1.25 | 0.013 |
| 18 | Ubiquitin-fold modifier 1 OS=Bos taurus OX=9913 GN=UFM1 PE=3 SV=1 | | Q2KJG2 | UFM1 | -0.77 | 0.013 |
| 19 | Isoamyl acetate-hydrolyzing esterase 1 homolog OS=Bos taurus OX=9913 GN=IAH1 PE=2 SV=1 | | Q3SZ16 | IAH1 | -0.83 | 0.015 |
| 20 | Cluster of Collagen type VI alpha 2 chain OS=Bos taurus OX=9913 GN=COL6A2 PE=1 SV=3 (F1MKG2) | | F1MKG2 | COL6A2 | -1.36 | 0.015 |
| 21 | Electron transfer flavoprotein subunit alpha OS=Bos taurus OX=9913 GN=ETFA PE=1 SV=1 | | F1MWR3 | ETFA | 0.58 | 0.016 |
| 22 | Pyruvate dehydrogenase E1 component subunit beta, mitochondrial OS=Bos taurus OX=9913 GN=PDHB PE=1 SV=2 | | P11966 | PDHB | 0.79 | 0.018 |
| 23 | HNRNPR protein OS=Bos taurus OX=9913 GN=HNRNPR PE=1 SV=1 | | A3KMV6 | HNRNPR | -0.45 | 0.019 |
| 24 | Mitotic checkpoint protein BUB3 OS=Bos taurus OX=9913 GN=BUB3 PE=2 SV=1 | | Q1JQB2 | BUB3 | -1.00 | 0.02 |
| 25 | Cluster of 40S ribosomal protein S6 OS=Bos taurus OX=9913 GN=RPS6 PE=2 SV=1 (Q5E995) | | Q5E995 | RPS6 | 0.79 | 0.021 |
| 26 | Leucyl-tRNA synthetase OS=Bos taurus OX=9913 GN=LARS1 PE=3 SV=1 | | A0A3Q1N108 | LARS1 | 0.94 | 0.021 |
| 27 | Eukaryotic translation initiation factor 4B OS=Bos taurus OX=9913 GN=EIF4B PE=1 SV=1 | | A0A3Q1MRD9 | EIF4B | 1.26 | 0.023 |
| 28 | Cluster of Peroxiredoxin-4 OS=Bos taurus OX=9913 GN=PRDX4 PE=2 SV=1 (Q9BGI2) | | Q9BGI2 | PRDX4 | -0.61 | 0.023 |
| 29 | Cluster of Transforming protein RhoA OS=Bos taurus OX=9913 GN=RHOA PE=1 SV=1 (P61585) | | P61585 | RHOA | -0.96 | 0.023 |
| 30 | Cluster of 60S ribosomal protein L17 OS=Bos taurus OX=9913 GN=RPL17 PE=3 SV=1 (A0A452DJF6) | | A0A452DJF6 | RPL17 | 0.75 | 0.024 |
| 31 | Cluster of Cytosolic iron-sulfur assembly component 2B OS=Bos taurus OX=9913 GN=CIAO2B PE=3 SV=2 (E1BC22) | | E1BC22 | CIAO2B | 0.98 | 0.026 |
| 32 | Acetyl-CoA acetyltransferase, mitochondrial OS=Bos taurus OX=9913 GN=ACAT1 PE=2 SV=1 | | Q29RZ0 | ACAT1 | -0.80 | 0.026 |
| 33 | Ubiquitin-conjugating enzyme E2 N OS=Bos taurus OX=9913 GN=UBE2N PE=1 SV=1 | | A0A3Q1MW26 | UBE2N | 0.99 | 0.027 |
| 34 | Programmed cell death protein 5 OS=Bos taurus OX=9913 GN=PDCD5 PE=2 SV=3 | | Q2HJH9 | PDCD5 | -1.03 | 0.027 |
| 35 | Copine-1 OS=Bos taurus OX=9913 GN=CPNE1 PE=3 SV=1 | | A0A3Q1MVI8 | CPNE1 | -0.27 | 0.027 |
| 36 | Rho GDP-dissociation inhibitor 1 OS=Bos taurus OX=9913 GN=ARHGDIA PE=1 SV=3 | | P19803 | ARHGDIA | -0.58 | 0.027 |
| 37 | G protein pathway suppressor 1 OS=Bos taurus OX=9913 GN=GPS1 PE=3 SV=1 | | A0A3Q1ME43 | GPS1 | -1.39 | 0.027 |
| 38 | 60S ribosomal protein L36a OS=Bos taurus OX=9913 GN=RPL36A PE=3 SV=3 | | Q3SZ59 | RPL36A | 1.63 | 0.027 |
| 39 | Apolipoprotein D OS=Bos taurus OX=9913 GN=APOD PE=3 SV=3 | | F1MS32 | APOD | 1.12 | 0.027 |
| 40 | 60S ribosomal protein L36 OS=Bos taurus OX=9913 GN=RPL36 PE=3 SV=3 | | Q3T171 | RPL36 | 3.28 | 0.028 |
| 41 | SUMO-activating enzyme subunit 2 OS=Bos taurus OX=9913 GN=UBA2 PE=1 SV=1 | | A0A3Q1MG31 | UBA2 | -1.26 | 0.028 |
| 42 | N-alpha-acetyltransferase 15, NatA auxiliary subunit OS=Bos taurus OX=9913 GN=NAA15 PE=4 SV=2 | | F1N4V5 | NAA15 | 0.79 | 0.028 |
| 43 | Very-long-chain (3R)-3-hydroxyacyl-CoA dehydratase OS=Bos taurus OX=9913 GN=HACD2 PE=3 SV=1 | | A0A3Q1M1C6 | HACD2 | 0.90 | 0.028 |
| 44 | Eukaryotic peptide chain release factor subunit 1 OS=Bos taurus OX=9913 GN=ETF1 PE=2 SV=3 | | Q0VCX5 | ETF1 | -1.81 | 0.028 |
| 45 | Fibronectin OS=Bos taurus OX=9913 GN=FN1 PE=4 SV=1 | | G5E5A8 | FN1 | -1.70 | 0.029 |
| 46 | 60S ribosomal protein L35 OS=Bos taurus OX=9913 GN=RPL35 PE=3 SV=1 | | A0A3Q1M4X0 | RPL35 | 1.01 | 0.029 |
| 47 | Cluster of Uncharacterized protein OS=Bos taurus OX=9913 GN=LOC516355 PE=3 SV=3 (F1MYQ8) | | F1MYQ8 | LOC516355 | -1.43 | 0.029 |
| 48 | Endoplasmin OS=Bos taurus OX=9913 GN=HSP90B1 PE=2 SV=1 | | Q95M18 | HSP90B1 | -0.23 | 0.03 |
| 49 | Cluster of Glyceraldehyde-3-phosphate dehydrogenase OS=Bos taurus OX=9913 GN=GAPDH PE=1 SV=4 (P10096) | | P10096 | GAPDH | 0.48 | 0.03 |
| 50 | Desmocollin-3 OS=Bos taurus OX=9913 GN=DSC3 PE=4 SV=3 | | E1BB21 | DSC3 | 1.24 | 0.03 |
| 51 | Secretory carrier-associated membrane protein OS=Bos taurus OX=9913 GN=SCAMP2 PE=1 SV=1 | | A6QR35 | SCAMP2 | 1.18 | 0.031 |
| 52 | Alpha-2-macroglobulin OS=Bos taurus OX=9913 GN=A2M PE=1 SV=2 | | Q7SIH1 | A2M | -0.58 | 0.031 |
| 53 | Cluster of Sorting nexin 6 OS=Bos taurus OX=9913 GN=SNX6 PE=2 SV=1 (Q2KI35) | | Q2KI35 | SNX6 | -0.52 | 0.032 |
| 54 | COP9 signalosome complex subunit 4 OS=Bos taurus OX=9913 GN=COPS4 PE=2 SV=1 | | Q3SZA0 | COPS4 | 0.89 | 0.033 |
| 55 | Cysteine and histidine-rich domain-containing protein 1 OS=Bos taurus OX=9913 GN=CHORDC1 PE=2 SV=1 | | Q29RL2 | CHORDC1 | -1.06 | 0.033 |
| 56 | Glutathione peroxidase 7 OS=Bos taurus OX=9913 GN=GPX7 PE=2 SV=1 | | A6QLY2 | GPX7 | 1.36 | 0.034 |
| 57 | Progesterone receptor membrane component 2 OS=Bos taurus OX=9913 GN=PGRMC2 PE=1 SV=1 | | F6QJJ8 | PGRMC2 | 0.96 | 0.034 |
| 58 | 60S ribosomal protein L31 OS=Bos taurus OX=9913 GN=NPAS2 PE=3 SV=1 | | A0A452DIW3) | NPAS2 | 1.09 | 0.034 |
| 59 | DNA replication licensing factor MCM3 OS=Bos taurus OX=9913 GN=MCM3 PE=2 SV=1 | | A4FUD9 | MCM3 | -1.14 | 0.035 |
| 60 | Cluster of Serine/threonine-protein phosphatase 2A 65 kDa regulatory subunit A alpha isoform OS=Bos taurus OX=9913 GN=PPP2R1A PE=4 SV=1 (A0A3Q1LW84) | | A0A3Q1LW84 | PPP2R1A | 0.55 | 0.036 |
| 61 | Myosin regulatory light chain 12B OS=Bos taurus OX=9913 GN=MYL12B PE=2 SV=1 | | A4IF97 | MYL12B | -0.88 | 0.036 |
| 62 | 60S acidic ribosomal protein P1 OS=Bos taurus OX=9913 GN=RPLP1 PE=3 SV=1 | | Q56K14 | RPLP1 | -0.92 | 0.036 |
| 63 | Cluster of Sodium/potassium-transporting ATPase subunit alpha-1 OS=Bos taurus OX=9913 GN=ATP1A1 PE=1 SV=1 (Q08DA1) | | Q08DA1 | ATP1A1 | 0.91 | 0.036 |
| 64 | 39S ribosomal protein L12, mitochondrial OS=Bos taurus OX=9913 GN=MRPL12 PE=1 SV=1 | | Q7YR75 | MRPL12 | 1.46 | 0.036 |
| 65 | Cluster of cAMP-dependent protein kinase catalytic subunit alpha OS=Bos taurus OX=9913 GN=PRKACA PE=1 SV=3 (P00517) | | P00517 | PRKACA | -1.23 | 0.037 |
| 66 | NPC intracellular cholesterol transporter 1 OS=Bos taurus OX=9913 GN=NPC1 PE=3 SV=1 | | A0A3Q1LT67 | NPC1 | 1.61 | 0.037 |
| 67 | RALY heterogeneous nuclear ribonucleoprotein OS=Bos taurus OX=9913 GN=RALY PE=2 SV=1 | | Q5E952 | RALY | -1.46 | 0.037 |
| 68 | Collagen type VI alpha 1 chain OS=Bos taurus OX=9913 GN=COL6A1 PE=1 SV=1 | | E1BI98 | COL6A1 | -0.49 | 0.037 |
| 69 | Calcyclin-binding protein OS=Bos taurus OX=9913 GN=CACYBP PE=2 SV=1 | | Q3T168 | CACYBP | 2.71 | 0.038 |
| 70 | Muscleblind like splicing regulator 1 OS=Bos taurus OX=9913 GN=MBNL1 PE=4 SV=1 | | A0A3Q1LJR6 | MBNL1 | 1.35 | 0.039 |
| 71 | Myotrophin OS=Bos taurus OX=9913 GN=MTPN PE=1 SV=3 | | Q3T0F7 | MTPN | -0.44 | 0.039 |
| 72 | 60S ribosomal protein L11 OS=Bos taurus OX=9913 GN=RPL11 PE=2 SV=3 | | Q3T087 | RPL11 | 0.70 | 0.04 |
| 73 | Phosphoserine aminotransferase OS=Bos taurus OX=9913 GN=PSAT1 PE=1 SV=1 | | A0A3S5ZPF7 | PSAT1 | -0.87 | 0.04 |
| 74 | Thiopurine S-methyltransferase OS=Bos taurus OX=9913 GN=TPMT PE=2 SV=1 | | Q17QQ2 | TPMT | 1.22 | 0.044 |
| 75 | Ribosomal protein L15 OS=Bos taurus OX=9913 PE=3 SV=1 | | A0A3Q1LST0 | RPL15 | 0.97 | 0.045 |
| 76 | Phosphatidylinositol transfer protein beta isoform OS=Bos taurus OX=9913 GN=PITPNB PE=4 SV=1 | | A0A452DJF3 | PITPNB | 1.13 | 0.047 |
| 77 | Cluster of Actin related protein 1A OS=Bos taurus OX=9913 GN=ACTR1A PE=1 SV=1 (F2Z4F0) | | F2Z4F0 | ACTR1A | -0.43 | 0.047 |
| 78 | Laminin subunit gamma 1 OS=Bos taurus OX=9913 GN=LAMC1 PE=1 SV=2 | | F1MD77 | LAMC1 | 1.58 | 0.048 |
| 79 | Dynactin subunit 2 OS=Bos taurus OX=9913 GN=DCTN2 PE=1 SV=1 | | A0A3Q1LSJ7 | DCTN2 | -0.95 | 0.048 |
| 80 | Cluster of Oxoglutarate dehydrogenase (succinyl-transferring) OS=Bos taurus OX=9913 GN=OGDH PE=1 SV=1 (A0A3Q1NKS6) | | A0A3Q1NKS6 | OGDH | -1.44 | 0.049 |

| **B** | |  | | | | |
| --- | --- | --- | --- | --- | --- | --- |
| **No** | **Protein** | | **Accession Number** | **Alternate ID** | **Log2**  **(Fold change)** | **T-TEST**  **(*P*-value)** |
| 1 | THO complex subunit 2 OS=Bos taurus OX=9913 GN=THOC2 PE=3 SV=3 | | F1N153 | THOC2 | -2.14 | 0.00031 |
| 2 | RNA-binding motif protein, X chromosome OS=Bos taurus OX=9913 GN=RBMX PE=1 SV=1 | | D3JUI8 | RBMX | -1.71 | 0.0016 |
| 3 | ATP synthase subunit d, mitochondrial OS=Bos taurus OX=9913 GN=ATP5PD PE=1 SV=2 | | P13620 | ATP5PD | 2.38 | 0.0031 |
| 4 | Tripeptidyl-peptidase 2 OS=Bos taurus OX=9913 GN=TPP2 PE=4 SV=1 | | A0A3Q1M996 | TPP2 | -0.77 | 0.004 |
| 5 | 60S ribosomal protein L30 OS=Bos taurus OX=9913 GN=RPL30 PE=3 SV=3 | | Q3T0D5 | RPL30 | -1.61 | 0.0043 |
| 6 | 40S ribosomal protein S27 OS=Bos taurus OX=9913 PE=3 SV=1 | | E1B7A8 | RPS27 | -1.23 | 0.0061 |
| 7 | Cluster of Heat shock 70 kDa protein 1B OS=Bos taurus OX=9913 GN=HSPA1B PE=2 SV=1 (Q27965) | | Q27965 | HSP70 | 2.50 | 0.0072 |
| 8 | Cluster of Guanine nucleotide-binding protein G(s) subunit alpha isoforms short OS=Bos taurus OX=9913 GN=GNAS PE=1 SV=1 (P04896) | | P04896 | GNAS | 0.73 | 0.0084 |
| 9 | B-cell receptor-associated protein OS=Bos taurus OX=9913 GN=BCAP31 PE=2 SV=1 | | Q5E9F1 | BCAP31 | -1.63 | 0.0085 |
| 10 | ATP synthase subunit alpha OS=Bos taurus OX=9913 GN=ATP5F1A PE=1 SV=1 | | F1MLB8 | ATP5F1A | 0.73 | 0.011 |
| 11 | CYB5B protein OS=Bos taurus OX=9913 GN=CYB5B PE=2 SV=2 | | Q0P5F6 | CYB5B | 0.99 | 0.013 |
| 12 | Synaptobrevin homolog YKT6 OS=Bos taurus OX=9913 GN=YKT6 PE=2 SV=1 | | Q3T000 | YKT6 | 1.44 | 0.014 |
| 13 | Septin-7 OS=Bos taurus OX=9913 GN=SEPTIN7 PE=3 SV=1 | | A0A3Q1M3S4 | SEPTIN7 | 1.40 | 0.019 |
| 14 | Cluster of ATP synthase subunit f, mitochondrial OS=Bos taurus OX=9913 GN=ATP5MF PE=1 SV=3 (Q28851) | | Q28851 | ATP5MF | 1.19 | 0.02 |
| 15 | Cluster of RAB1A, member RAS oncogene family OS=Bos taurus OX=9913 GN=RAB1A PE=1 SV=1 (A1L528) | | A1L528 | RAB1A | 0.51 | 0.021 |
| 16 | Interleukin enhancer binding factor 2 OS=Bos taurus OX=9913 GN=ILF2 PE=4 SV=1 | | A0A3Q1NGX5 | ILF2 | -0.74 | 0.022 |
| 17 | Cluster of ADP/ATP translocase 3 OS=Bos taurus OX=9913 GN=SLC25A6 PE=1 SV=3 (P32007) | | P32007 | SLC25A6 | 0.63 | 0.023 |
| 18 | Cysteine-rich protein 2 OS=Bos taurus OX=9913 GN=CRIP2 PE=2 SV=1 | | Q0VFX8 | CRIP2 | -2.04 | 0.024 |
| 19 | Peroxiredoxin-5, mitochondrial OS=Bos taurus OX=9913 GN=PRDX5 PE=2 SV=2 | | Q9BGI1 | PRDX5 | 1.16 | 0.025 |
| 20 | LDL receptor related protein associated protein 1 OS=Bos taurus OX=9913 GN=LRPAP1 PE=1 SV=1 | | A0A3Q1MD85 | LRPAP1 | -0.97 | 0.026 |
| 21 | Septin-2 OS=Bos taurus OX=9913 GN=SEPTIN2 PE=2 SV=1 | | Q2NKY7 | SEPTIN2 | 0.89 | 0.029 |
| 22 | Cluster of Heat shock protein HSP 90-alpha OS=Bos taurus OX=9913 GN=HSP90AA1 PE=1 SV=3 (Q76LV2) | | Q76LV2 | HSP90 | 1.31 | 0.031 |
| 23 | Acetyl-CoA acyltransferase 1 OS=Bos taurus OX=9913 GN=ACAA1 PE=1 SV=1 | | Q3ZC41 | ACAA1 | -2.57 | 0.031 |
| 24 | Programmed cell death 6 interacting protein OS=Bos taurus OX=9913 GN=PDCD6IP PE=1 SV=3 | | E1BKM4 | PDCD6IP | -0.81 | 0.032 |
| 25 | Cluster of Collagen type VI alpha 2 chain OS=Bos taurus OX=9913 GN=COL6A2 PE=1 SV=3 (F1MKG2) | | F1MKG2 | COL6A2 | -0.91 | 0.036 |
| 26 | 40S ribosomal protein S30 OS=Bos taurus OX=9913 PE=3 SV=1 | | A0A3Q1MG78 | RPS30 | 0.87 | 0.036 |
| 27 | BAG cochaperone 3 OS=Bos taurus OX=9913 GN=BAG3 PE=1 SV=1 | | F1MIU2 | BAG3 | 1.77 | 0.037 |
| 28 | RALY heterogeneous nuclear ribonucleoprotein OS=Bos taurus OX=9913 GN=RALY PE=2 SV=1 | | Q5E952 | RALY | -1.45 | 0.037 |
| 29 | Acyl-CoA-binding protein OS=Bos taurus OX=9913 GN=DBI PE=1 SV=2 | | P07107 | DBI | -0.76 | 0.037 |
| 30 | 40S ribosomal protein S23 OS=Bos taurus OX=9913 GN=RPS23 PE=2 SV=1 | | Q3T199 | RPS23 | -1.93 | 0.039 |
| 31 | Coronin OS=Bos taurus OX=9913 GN=CORO1C PE=2 SV=1 | | A2VDN8 | CORO1C | -0.94 | 0.04 |
| 32 | Golgi phosphoprotein 3 OS=Bos taurus OX=9913 GN=GOLPH3 PE=2 SV=1 | | Q1RMW9 | GOLPH3 | 1.86 | 0.04 |
| 33 | Ras suppressor protein 1 OS=Bos taurus OX=9913 GN=RSU1 PE=4 SV=1 | | A0A3Q1M5B4 | RSU1 | 0.63 | 0.042 |
| 34 | Annexin OS=Bos taurus OX=9913 GN=ANXA3 PE=3 SV=1 | | F1MWQ2 | ANXA3 | 0.50 | 0.042 |
| 35 | ATP synthase subunit beta, mitochondrial OS=Bos taurus OX=9913 GN=ATP5F1B PE=1 SV=2 | | P00829 | ATP5F1B | 0.69 | 0.042 |
| 36 | Apolipoprotein D OS=Bos taurus OX=9913 GN=APOD PE=3 SV=3 | | F1MS32 | APOD | 1.19 | 0.043 |
| 37 | Cluster of Ras-related protein Rab-5C OS=Bos taurus OX=9913 GN=RAB5C PE=1 SV=1 (A0A3Q1N8E3) | | A0A3Q1N8E3 | RAB5C | 0.93 | 0.043 |
| 38 | N-alpha-acetyltransferase 10 OS=Bos taurus OX=9913 GN=NAA10 PE=2 SV=1 | | Q2KI14 | NAA10 | -1.24 | 0.044 |
| 39 | Heteroous nuclear ribonucleoprotein D like OS=Bos taurus OX=9913 GN=HNRNPDL PE=1 SV=1 | | A0A3Q1NLI5 | HNRNPDL | -1.05 | 0.045 |
| 40 | Proteasome subunit alpha type-6 OS=Bos taurus OX=9913 GN=PSMA6 PE=1 SV=1 | | Q2YDE4 | PSMA6 | 1.49 | 0.045 |
| 41 | Transgelin OS=Bos taurus OX=9913 GN=TAGLN PE=1 SV=4 | | Q9TS87 | TAGLN | -0.18 | 0.045 |
